# Relationships between Anxiety, Depression, and Illness Perceptions in Lung and Breast Cancer Patients throughout the Cancer Continuum

**DOI:** 10.3390/healthcare11202794

**Published:** 2023-10-22

**Authors:** Burcu Sırlıer Emir, Sevler Yıldız, Osman Kurt, Elif Emre, Süleyman Aydın

**Affiliations:** 1 Department of Psychiatry, Elazığ Fethi Sekin City Hospital, 23100 Elazığ, Turkey; sevler.yildiz@saglik.gov.tr; 2 Department of Public Health, Adıyaman Provincial Health Directorate, 02100 Adıyaman, Turkey; osman.kurt2@saglik.gov.tr; 3 Department of Anatomy, Faculty of Medicine, University of Fırat, 23119 Elazığ, Turkey; eemre@firat.edu.tr; 4 Department of Biochemistry, Faculty of Medicine, University of Fırat, 23119 Elazığ, Turkey; saydin1@firat.edu.tr

**Keywords:** cancer, illness perception, depression, anxiety

## Abstract

Cancer is a devastating disease that has significant psychological and biological impacts. Generally, lung cancer primarily affects men while breast cancer primarily affects women. Thus, this study aimed to investigate the levels of anxiety and depression in patients with these prevalent cancer types, as well as their perceptions of the illness and any potential connections between them. The study included a total of 252 participants, consisting of 110 breast cancer patients, 112 lung cancer patients, and 30 healthy individuals as controls. The Beck Depression Inventory (BDI) and Beck Anxiety Inventory (BAI) were administered to assess mood, while the Illness Perception Questionnaire (IPQ) was used to evaluate cancer perceptions. Results revealed that both breast cancer and lung cancer patients had significantly higher BDI and BAI scores compared to the control group. Furthermore, the BDI and BAI scores were lower in breast cancer patients compared to lung cancer patients. The IPQ causal representation–immunity score was significantly higher in lung cancer patients than in breast cancer patients (*p* = 0.01). Positive correlations were found between BDI scores and BAI scores, as well as between BDI scores and certain subscale scores of the IPQ related to illness representation and causal representation. Additionally, a positive correlation was observed between BAI scores and the IPQ illness representation–timeline acute/chronic subscale, while a negative correlation was found between BAI scores and the IPQ causal representation–accident or chance scores. Overall, the study findings demonstrated that breast and lung cancer patients possess negative perceptions of their disease and experience high levels of anxiety and depression. To enhance the quality of life and promote resilience in these patients, it is recommended to incorporate psychological interventions that consider anxiety, depression, and disease perception.

## 1. Introduction

Cancer is a significant global health issue that requires comprehensive consideration of its physical, psychological, and social aspects [1,2]. Recently, the incidence of newly diagnosed cases of lung and breast cancer worldwide has surpassed 1.7 million and 2 million, respectively [1,3]. Alongside the treatments for these diseases, individuals diagnosed with cancer often face psychological challenges when coping with physical symptoms and adjusting to new circumstances [4,5]. Feelings of anxiety, rebellion, and fear may arise in response to the cancer diagnosis and the subsequent treatment process [5]. Depression and anxiety disorders are commonly found in cancer patients [6,7]. Pitman et al. report a higher susceptibility to these mental health conditions in cancer patients compared to the general population [8]. Additionally, studies have revealed that 8–16% of cancer patients experience depressive disorders [9], and 19% experience anxiety disorders [10]. There are neural substrates or underlying neurological mechanisms underlying these mental problems such as depression and anxiety experienced by cancer patients. Anxiety is known to affect cognitive structures such as the prefrontal cortex, amygdala, and hippocampus at high levels. The prefrontal cortex and hypothalamic–pituitary–adrenal (HPA) axis play a role in emotional regulation and stress response [11,12,13]. The co-existence of these mental disorders with cancer may affect the individual’s perception of the disease and may lead to negative effects on treatment compliance, quality of life, and prognosis [14,15].

The relationships between depression, anxiety, and illness perceptions are well-established across a range of physical illnesses [16,17]. Zhu et al. proposed a close association between illness perceptions and psychological symptoms in cancer patients [18]. Another study demonstrated that patients with gastrointestinal cancers who perceived cancer negatively experienced elevated levels of stress [19]. However, in our investigation, we could not find any studies that assessed these factors concurrently in both lung and breast cancer patients, two of the most prevalent types of cancer. Therefore, the aim of our study was to investigate the relationship between depression, anxiety, and illness perception in individuals diagnosed with breast cancer and lung cancer, which are the most common types of cancer. We think that determining the mental status of these patients and making the necessary psychiatric interventions may have a positive effect on the treatment process of cancer disease.

## 2. Materials and Methods

### 2.1. Participants and Procedure

Approval from the local ethics committee at Fırat University Faculty of Medicine (Approval No.: 2022/12-10) was obtained for this study. This study was conducted between November 2022 and August 2023 at Fethi Sekin City Hospital, especially in the Mental Health and Diseases Outpatient Clinic. In accordance with the ethical standards outlined in the 1983 revision of the Declaration of Helsinki, participants diagnosed with breast cancer and lung cancer were randomly selected for the study. The participants consisted of individuals receiving active treatment as well as those who had completed treatment and were undergoing routine check-ups. Selection criteria included age (participants had to be older than 18 years), histologically diagnosed cancer, absence of cognitive or neurological impairments that could interfere with answering questions, absence of known psychiatric conditions, and voluntary participation in the study. The exclusion criteria were as follows: decreased cognitive function, having cancer other than breast or lung cancer, and being younger than 18 years of age, illiterate, and volunteer to participate in the study. A total of 232 previously diagnosed cancer patients who met the inclusion criteria and were either receiving outpatient or inpatient treatment or in remission, along with 30 healthy controls who did not have any mental disorders according to DSM-5, were included in the study. The patient group was divided into two subgroups based on their cancer type: breast cancer and lung cancer. A psychiatrist conducted structured interviews with all participants according to DSM-5, with each interview lasting approximately 30 min. After written informed consent was obtained from all participants, the Beck Depression Inventory, Beck Anxiety Inventory, and Illness Perception Questionnaire, which are widely accepted and applicable in psychological research, were completed.

### 2.2. Data Collection Tools

#### 2.2.1. Sociodemographic Data Form

This form included questions concerning demographic information, such as age, marital status, and place of residence, as well as clinical evaluation questions related to disease diagnosis, treatment history, and use of substances like smoking or alcohol.

#### 2.2.2. Beck’s Depression Inventory (BDI)

The BDI, developed by Beck [20] to assess the level of depression, underwent a Turkish validity and reliability study conducted by Hisli [21]. In this study, the Cronbach alpha reliability coefficient value of the scale was 0.85.

#### 2.2.3. Beck’s Anxiety Inventory (BAI)

The original scale initially was designed by Beck [22]. A Turkish validity and reliability study was conducted by Ulusoy et al. [23]. In this study, the Cronbach alpha reliability coefficient value of the scale was 0.87.

#### 2.2.4. Illness Perception Questionnaire (IPQ)

The IPQ, created by Weinman et al. [24] and revised by Moss-Morris [25], was utilized to assess illness perception. Armay et al. [26] conducted the Turkish validity and reliability study. As the illness perception score increases, individuals tend to be more affected by the disease according to various parameters and perceive the disease as more worrisome. IPQ consists of three dimensions: identity, illness representation, and causal representation. The illness identity dimension includes common disease symptoms. It is divided into two subscales: Identity A, which represents patients experiencing various symptoms, and Identity B, which reflects patients perceiving their symptoms to be related to their illness. A higher score in the illness identity B dimension indicates a strong belief that the patient’s symptoms are associated with the disease. The dimension of illness representation includes seven subscales: timeline (acute/chronic), consequences, personal control, treatment control, illness coherence, timeline (cyclical), and emotional representations. The timeline subscales explore the individual’s perceptions of the duration of their illness. A higher score in the timeline (acute/chronic) subscale indicates a chronic condition, while a higher score in the timeline (cyclical) subscale suggests a cyclic nature of the condition. The consequences subscale investigates beliefs about the severity and potential impact of the illness on physical, social, and psychological functioning. A higher score in the consequences subscale indicates negative consequences of the illness. Personal control examines individuals’ internal perceptions of control over the duration, course, and treatment of their illness. Treatment control explores beliefs about the effectiveness of the applied treatment. Higher scores in the personal control and treatment control subscales indicate positive beliefs about controlling the illness and treatment. The comprehension of illness coherence subscale reflects the individual’s ability to understand their condition, with higher scores indicating higher personal understanding. The emotional representations subscale assesses the increase in negative emotions associated with the illness. The dimension of causal representation investigates the individual’s thoughts about the possible causes of their illness and includes four subscales: psychological attributions, risk factors, immunity, and accident or chance. In the IPQ, the scores obtained from individual items are summed to form the subscale total. In this study, the identity dimension had values between 0.72–0.87, the illness representation dimension had a Cronbach’s alpha value of 0.68–0.88, and the causal representation dimension had values between 0.75–0.90.

### 2.3. Statistical Analysis

The data were analyzed in SPSS (Statistical Package for Social Sciences; SPSS Inc., Chicago, IL, USA) 22 package program. Descriptive statistics were presented as n and % values for categorical data and median (minimum-maximum) values for continuous data. Chi-square analysis (Pearson Chi-square) was used to compare categorical variables between groups. The conformity of continuous variables to normal distribution was evaluated by Kolmogorov–Smirnov test. Mann–Whitney U test was used to compare variables between two groups and the Kruskal Wallis test was used to compare variables between more than two groups to data that do not have normal distribution. Spearman correlation test was used to examine the relationship between continuous variables. Univariate logistic regression analysis and multivariate logistic regression analysis with a stepwise approach were performed to determine the risk of anxiety. Variables eligible for inclusion in the multivariate analysis were tested for collinearity. Variables that remained significant (*p* < 0.05) in the multivariate model were considered independent predictors of moderate–severe anxiety levels. Hosmer–Lemeshow goodness of fit statistics were performed to assess model fit. Odds ratios (ORs) and 95% confidence intervals (CIs) were calculated for each predictor. Statistical significance level was accepted as *p* < 0.05 in all analyses.

## 3. Results

A total of 252 participants were included in the study: 110 (36.7%) breast cancer patients, 112 (37.3%) lung cancer patients, and 30 (10%) healthy controls. Among the breast cancer patients, 99.1% were female, compared to 29.5% of lung cancer patients and 66.7% of healthy controls, resulting in a significant gender difference (*p* < 0.001). The mean age of lung cancer patients was higher than that of healthy controls, and there was a significant age difference between the groups (*p* = 0.012). Breast cancer patients had a significantly longer disease duration compared to lung cancer patients (*p* = 0.021). Alcohol consumption was found among 5.4% of lung cancer patients, while none of the breast cancer patients or healthy controls consumed alcohol (*p* = 0.021). Family history of cancer was present in 38.2% of breast cancer patients, 42% of lung cancer patients, and 16.7% of healthy controls, with a significant difference observed between the groups (*p* = 0.038). This difference was specific to lung cancer patients and healthy controls (Table 1).

Significant differences were found between the groups in terms of BDI (*p* < 0.001) and BAI (*p* = 0.007) scores, with both cancer types having significantly higher scores compared to healthy controls. The causal representation–immunity subscale score of IPQ was notably higher in the lung cancer group compared to the breast cancer group (*p* = 0.01) (Table 2).

A significant difference was observed in the illness representation–timeline acute/chronic subscale scores of IPQ based on cancer stage (*p* = 0.003), specifically between stage 1 patients and stage 2 and stage 3 patients. Likewise, there were significant differences between patients at stage 4 and those at stages 2 and 3 in scores on the illness representation–consequences subscale, with patients at stage 4 scoring highest. The illness representation–treatment control subscale scores of IPQ also exhibited a significant difference between the groups (*p* = 0.017), related to stage 4 patients and stage 2 and stage 3 patients. Additionally, a significant difference emerged in the illness representation-illness coherence subscale scores of IPQ (*p* = 0.023), primarily between stage 4 patients and stage 2 and stage 3 patients. The perceived illness representation–emotional representations subscale scores of IPQ also showed a significant difference between the groups (*p* < 0.001), again due to the contrast between stage 4 patients and stage 2 and stage 3 patients. Furthermore, a significant difference was observed in the causal representation-psychological attributions subscale scores of IPQ (*p* < 0.001), attributable to the difference between stage 1 patients and stage 2 and stage 3 patients, as well as stage 4 patients and stage 2 and stage 3 patients. The causal representation–risk factors subscale scores of IPQ exhibited a significant difference between the groups (*p* < 0.001), with stage 4 patients showing distinction from other stages. The causal representation–immunity subscale scores of IPQ also displayed a significant difference (*p* = 0.001), attributed to the contrast between stage 1 patients and stage 2 and stage 3 patients, as well as stage 4 patients and stage 2 and stage 3 patients (Table 3).

Significant positive correlations were observed between BDI scores and BAI scores (*p* < 0.01, r = 0.848), the illness representation–timeline acute/chronic subscale scores of IPQ (*p* = 0.032, r = 0.131), and the causal representation–risk factors subscale scores of IPQ (*p* = 0.001, r = 0.194). Furthermore, a significant positive correlation was found between BDI scores and the causal representation–psychological attributions subscale scores of IPQ (*p* = 0.005, r = 0.169). In addition, a significant positive correlation was observed between BAI scores and IPQ disease representation–timeline acute/chronic subscale scores (*p* = 0.026, r = 0.136), and a significant positive correlation was found between BAI scores and IPQ causal representation–accident or chance scores (*p* = 0.04, r = 0.125) (Table 4).

After patients were categorized into 2 groups according to Beck Depression Inventory scores (“mild” level of anxiety vs. “moderate–severe” level of anxiety), a binary logistic regression analysis was performed to detect the possible parameters that affect moderete–severe anxiety level. Logistic regression analysis demonstrated that higher Beck Depression Inventory and IPQ-illness representation–emotional representations scores were independently related to increased anxiety levels. The results of logistic regression analysis are summarized in Table 5.

## 4. Discussion

In the present study, it was observed that both breast cancer and lung cancer patients had higher levels of depression and anxiety symptoms compared to the healthy control group. Additionally, it was found that illness perception was elevated in both cancer types, and as the illness perception score increased, depression and anxiety levels also increased. Upon examining the illness perception level of the patients, it was discovered that stage 1 patients had lower scores in the timeline acute/chronic sub-dimension compared to patients in other stages. This sub-dimension refers to how patients perceive the duration of the disease and indicates that patients in advanced stages perceive the disease as permanent, whereas those in the early stages see it as temporary and do not fully accept the disease. The findings of our study show that both lung and breast cancer patients developed the belief that the disease is permanent at stage 1 and stage 4, which is in line with expectations. In a previous study on chronic diseases, it was reported that viewing the disease as chronic enhances control over it [27,28]. Although no significant difference was found in the consequences sub-dimension of the illness perception questionnaire (IPQ) between cancer types, a significant difference was observed between cancer stages. The consequences sub-dimension pertains to how patients perceive the psychological and physical effects of the disease and suggest that acceptance becomes more prominent as the disease progresses towards the terminal phase. In contrast to our findings, a study conducted in 2021 on lymphoma patients revealed that patients in the early stage had a positive outlook on their disease [29]. This suggests that following a cancer diagnosis, regardless of the cancer type, the perception that cancer is an incurable and fatal illness dominates society. While the treatment control sub-dimension score did not differ between cancer types, it was higher in stage 4 patients compared to stage 2 and 3 patients. This sub-dimension refers to the effectiveness and controllability of the treatment. Zang et al. also found a high treatment control sub-score in cancer patients [30]. Although end-stage patients are typically expected to have a low belief in their ability to control the disease, a high treatment control subscale score may be interpreted as a high belief in treatment in terminally ill patients [25,31]. Illness coherence sub-dimension scores also varied between cancer stages. In the final stage, it can be suggested that patients are capable of making sense of the disease or are well-informed due to having experienced every stage of the disease and lived with it for a longer duration. Studies conducted with cancer patients have reported both high [32] and low [33] illness coherence scores. It was found that patients who believed they did not receive sufficient information after diagnosis had more negative illness perceptions [34]. In the present study, the emotional representations sub-dimension score was higher in stage 4 patients.

A study carried out on breast cancer patients found high emotional representation scores and stated that patients experienced intense negative emotions regarding the illness [35]. In line with this study, we also found that emotional representation scores were independently related to increased anxiety levels. There was a significant difference between cancer stages in the psychological attributions sub-dimension, and a positive correlation was observed with the Beck Depression Inventory (BDI). The reason for the high scores in the initial stage may be that patients examined the causes of the disease from the moment they learned about it, particularly attributing psychological reasons to the cause of the disease. The reason for the high scores in the final stage may be the concern that their life will end and the feeling of being unable to overcome feelings of helplessness, which could explain the high scores in the psychological factors subscales.

Patients in stage 4 exhibited lower scores in the risk factors sub-dimension compared to patients in other stages, and there was a positive correlation between scores on the Beck Anxiety Inventory (BAI) and Beck Depression Inventory (BDI) [35]. This suggests that in the early stages, cancer patients tend to attribute risk factors such as malnutrition and bad habits as causes of the disease. A study conducted on cancer patients found that they primarily perceived risk factors as the causes of their illness. Interestingly, despite the literature suggesting that lung cancer may have higher risk factor scores due to factors like environmental pollution and smoking, there was no significant difference in risk factor scores between lung cancer and breast cancer. However, in terms of immunity, patients in stage 2 and stage 3 had lower scores, and lung cancer patients had higher immunity scores compared to breast cancer patients. Another study on older cancer patients found that immunity was the most significant cause of the disease [36]. In cancer interventions, it is important to understand patients’ perceptions of the disease, identify their negative evaluations attributed to the illness, and effectively address these issues during the treatment process. Specifically, various cognitive behavioral therapy programs have been developed for cancer patients with specific psychiatric disorders and disease groups [37,38]. However, research suggests that while these therapies are effective in the early stages of the disease, they may not be sufficiently helpful for patients in the advanced stage [39]. We believe that evaluating patients’ perceptions of their illness in lung and breast cancer can provide guidance for implementing psychological interventions at different stages of the cancer journey.

The coexistence of depression and anxiety with cancer is emphasized in numerous studies [37,38,40]. This study also found that depression and anxiety were significantly more prevalent in cancer patients compared to healthy controls. It is not surprising that anxiety is common in cancer, as it is an unknown and uncertain disease. Ferrario et al. found that cancer patients have high levels of trait anxiety [41]. Additionally, it has been noted that the rate of depression is highest in breast cancer patients within the first year of diagnosis [42]. Negative perceptions of illness are also linked to depression and anxiety [43,44]. Similar to our findings, a study comparing different types of cancer found no difference in depression levels [45], whereas another study reported an increased rate of depression in breast cancer patients [46]. Although we did not find a difference in depression and anxiety levels based on cancer stages, another study found higher levels of anxiety and depression in the later stages compared to the earlier stages [47]. Furthermore, patients with comorbid depression and cancer experience more severe symptoms such as anxiety, pain, fatigue, and decreased functionality, along with an increased risk of suicidal thoughts [48].

In terms of patient demographics, the mean age of lung cancer patients in this study was higher than that of breast cancer patients and healthy controls. The existing literature also indicates that the majority of lung cancer cases occur in individuals over the age of 50 [49]. Similarly, a majority of the lung cancer patients in this study were male, while a majority of the breast cancer patients were female, which aligns with previous research [50,51]. A significant difference was observed in terms of family history of cancer between the lung cancer group and the control group. Similarly, studies on lung cancer have also suggested a genetic predisposition to the disease [52]. While no difference in family history of cancer was detected in the breast cancer group, other studies have indicated that a familial predisposition is the most significant risk factor for developing breast cancer [53].

Despite the common occurrence of mental disorders in cancer patients, they are often disregarded and left untreated [54]. Psychological interventions such as cognitive–behavioral therapy and group therapies have been shown to have positive effects on breast cancer patients [55]. Psycho-oncologic approaches are also an important component of oncologic rehabilitation, aiming to address false beliefs about the disease, discuss anxieties and fears, help patients adjust to new conditions brought on by the illness, and assist patients in coping with the emotional impact of the disease.

Another key finding is that we found that in lung and breast cancer patients depression is a predictor of anxiety as a result of binary logistic regression. Consistent with our study, studies investigating the relationship between anxiety, depression, and disease perception also reported similar findings [56,57].

There are specific constraints inherent to the current study. Due to its cross-sectional nature and single-center design, it is impractical to extrapolate the obtained results to a broader context. It is a limitation that two different diseases were analyzed in the patients included in this study. The type of cancer may influence the psychological response to the disease. Therefore, the lack of a homogenous sample population represents an important limitation of this study. Nevertheless, our study possesses strengths through its considerable sample size.

## 5. Conclusions

In the present study, it was observed that both breast cancer and lung cancer patients had higher levels of depression and anxiety symptoms compared to the healthy control group. Additionally, it was found that illness perception was elevated in both cancer types, and as the illness perception score increased, depression and anxiety levels also increased. The results obtained in this study indicate that cancer patients have unfavorable perceptions about their illness and experience anxiety and depression. Previous research has found a correlation between negative illness perception and depression and anxiety symptoms in skin and breast cancer patients [44,58]. However, cancer patients are known to use antidepressants to cope with these mental symptoms [59]. We believe that offering cognitive and, if necessary, pharmacological interventions to cancer patients following their diagnosis will assist them in accepting their condition and employing effective coping mechanisms. This, in turn, will enable their active participation in treatment. Our findings highlight the advantages of assessing the mental well-being of breast cancer and lung cancer patients in psychiatric clinics using the Beck Depression Scale, the Beck Anxiety Scale, and the Illness Perception Questionnaire, as they can greatly enhance their quality of life. These scales will enable the mental status of lung and breast cancer patients to be recognized. It will be a guide for early psychiatric intervention in necessary patients. Furthermore, we propose that expanding the screening process to include other cancer types, alongside breast and lung cancers, will also contribute to improving patients’ quality of life and alleviate the burden on their families.

## Figures and Tables

**Table 1:** Comparison of all characteristics according to groups.

		Healthy Control		Breast Cancer		Lung Cancer		*p*
		Number	%	Number	%	Number	%	
Sex	Female	20	66.7 ^c^	109	99.1 ^a^	33	29.5 ^b^	0.001 *
	Male	10	33.3	1	0.9	79	70.5	
Age, Median (min-max)		56.5 (39.0–73.0) ^b^		62.0 (27.0–89.0) ^a,b^		65.0 (29.0–89.0) ^a^		0.012 **
Marital status	Single	5	16.7	26	23.6	25	22.3	0.718 *
	Married	25	83.3	84	76.4	87	77.7	
Education level	Primary school and below	8	26.7	62	56.4	52	46.4	0.068 *
	Middle school	10	33.3	20	18.2	26	23.2	
	High school and above	12	40.0	28	25.5	34	30.4	
Place of residence	District/village	7	23.3	31	28.2	33	29.5	0.803 *
	Province	23	76.7	79	71.8	79	70.5	
Income status	Poor	6	20.0	35	31.8	26	23.2	0.197 *
	Moderate	20	66.7	67	60.9	81	72.3	
	Good	4	13.3	8	7.3	5	4.5	
Employment status	Working	12	40.0	23	20.9	29	25.9	0.102 *
	Not working	18	60.0	87	79.1	83	74.1	
Comorbid organic disease	Yes	19	63.3	60	54.5	64	57.1	0.686 *
	No	11	36.7	50	45.5	48	42.9	
Duration of cancer diagnosis, Median (min-max)		-		3.0 (1.0–28.0%)		2.0 (1.0–19.0%)		0.021 ***
Cancer stage	Stage 1	-		25	22.7	25	22.3	1.000 *
	Stage 2			30	27.3	31	27.7	
	Stage 3			30	27.3	31	27.7	
	Stage 4			25	22.7	25	22.3	
Psychiatric treatment before diagnosis	Yes	-		22	20.0	15	13.4	0.187 *
	No			88	80.0	97	86.6	
Psychiatric treatment after diagnosis	Yes	-		35	31.8	36	32.1	0.959 *
	No			75	68.2	76	67.9	
Benefit from psychiatric treatment	Yes	-		26	23.6	26	23.2	0.941 *
	No			84	76.4	86	76.8	
History of surgery	Yes	-		78	70.9	77	68.8	0.726 *
	No			32	29.1	35	31.3	
History of chemotherapy	Yes	-		78	70.9	79	70.5	0.951 *
	No			32	29.1	33	29.5	
History of radiotherapy	Yes	-		77	70.0	70	62.5	0.237 *
	No			33	30.0	42	37.5	
Smoking	Yes	7	23.3	29	26.4	28	25.0	0.937 *
	No	23	76.7	81	73.6	84	75.0	
Alcohol use	Yes	0	0 ^a^	0	0 ^a^	6	5.4 ^b^	0.021 *
	No	30	100.0	110	100.0	106	94.6	
Other cancer type	Yes	-		10	9.1	8	7.1	0.595 *
	No			100	90.9	104	92.9	
Relapse	Yes	-		12	10.9	5	4.5	0.071 *
	No			98	89.1	107	95.5	
Hospitalization for cancer	Yes	-		84	76.4	93	83.0	0.216 *
	No			26	23.6	19	17.0	
Family history of cancer	Yes	5	16.7 ^b^	42	38.2 ^a,b^	47	42.0 ^a^	0.038 *
	No	25	83.3	68	61.8	65	58.0	
Loss of a loved one due to cancer	Yes	3	10.0	33	30.0	35	31.3	0.061 *
	No	27	90.0	77	70.0	77	68.8	

* Chi-square analysis, ** Kruskal–Wallis test, *** Mann–Whitney U test was applied. ^a,b,c^ Group where the difference originated.

**Table 2:** Comparison of scale scores according to groups.

	Healthy Control	Breast Cancer	Lung Cancer	*p*
	Median (Min–Max)	Median (Min–Max)	Median (Min–Max)	
BDI	3.0 (0.0–14.0) ^b^	7.0 (0.0–41.0) ^a^	9.0 (0.0–32.0) ^a^	<0.001 *
BAI	4.0 (0.0–14.0) ^b^	7.0 (0.0–35.0) ^a^	8.0 (0.0–30.0) ^a^	0.007
IPQ-Identity A		8.0 (1.0–14.0%)	8.0 (2.0–14.0%)	0.620 **
IPQ-Identity B		6.0 (1.0–14.0%)	5.5 (1.0–19.0%)	0.967
IPQ-Illness representation–Timeline acute/chronic		17.0 (7.0–28.0%)	16.0 (8.0–27.0%)	0.265
IPQ-Illness representation–Consequences		21.0 (10.0–33.0%)	20.5 (12.0–34.0%)	0.796
IPQ-Illness representation–Personal Control		19.5 (10.0–31.0%)	19.0 (10.0–34.0%)	0.139
IPQ-Illness representation–Treatment Control		14.0 (3.0–24.0%)	14.0 (6.0–23.0%)	0.234
IPQ-Illness representation–Illness Coherence		11.0 (4.0–21.0%)	10.0 (4.0–22.0%)	0.834 **
IPQ-Illness representation–Timeline cyclical		15.0 (6.0–20.0%)	16.0 (6.0–20.0%)	0.158
IPQ-Illness representation–Emotional Representations		18.0 (8.0–34.0%)	18.0 (10.0–32.0%)	0.355
IPQ-Causal representation–Psychological Attributions		16.0 (8.0–24.0%)	15.0 (8.0–23.0%)	0.284
IPQ-Causal representation–Risk Factors		16.0 (10.0–25.0%)	16.0 (8.0–23.0%)	0.334
IPQ-Causal representation–Immunity		6.0 (3.0–12.0%)	7.0 (3.0–14.0%)	0.010
IPQ-Causal representation–Accident or Chance		7.0 (2.0–10.0%)	6.0 (2.0–10.0%)	0.217

* Kruskal–Wallis and ** Mann–Whitney U tests were applied. ^a,b^ Group where the difference originated.

**Table 3:** Comparison of scale scores according to cancer stage.

	Stage 1	Stage 2	Stage 3	Stage 4	*p* *
	Median (Min–Max)	Median (Min–Max)	Median (Min–Max)	Median (Min–Max)	
BDI	8.0 (0–32.0%)	11.0 (0–41.0%)	9.0 (0–29.0%)	7.0 (0–35.0%)	0.640
BAI	8.0 (0–30.0%)	8.0 (0–29.0%)	7.0 (0–35.0%)	4.5 (0–29.0%)	0.462
IPQ-Identity A	8.0 (2.0–12.0%)	8.0 (2.0–14.0%)	8.0 (1.0–14.0%)	8.0 (4.0–13.0%)	0.706
IPQ-Identity B	6.0 (1.0–19.0%)	6.0 (1.0–14.0%)	5.0 (1.0–12.0%)	6.0 (4.0–14.0%)	0.319
IPQ-Illness representation–Timeline acute/chronic	14.5 (9.0–23.0) ^a^	18.0 (7.0–27.0) ^b^	18.0 (7.0–28.0) ^b^	15.0 (8.0–27.0) ^a,b^	0.003
IPQ-Illness representation–Consequences	22.0 (11.0–32.0) ^a,b^	20.0 (10.0–32.0) ^a^	19.0 (12.0–32.0) ^a^	23.0 (14.0–34.0) ^b^	0.029
IPQ-Illness representation–Personal Control	18.5 (10.0–31.0%)	18.0 (12.0–31.0%)	19.0 (10.0–34.0%)	20.0 (10.0–33.0%)	0.545
IPQ-Illness representation–Treatment Control	13.5 (6.0–23.0) ^a,b^	13.0 (6.0–24.0) ^a^	14.0 (3.0–24.0) ^a^	16.0 (7.0–23.0) ^b^	0.017
IPQ-Illness representation–Illness Coherence	11.0 (4.0–21.0) ^a,b^	10.0 (5.0–19.0) ^a^	10.0 (4.0–21.0) ^a^	13.0 (5.0–22.0) ^b^	0.023
IPQ-Illness representation–Timeline Cyclical	15.5 (6.0–20.0%)	16.0 (8.0–20.0%)	16.0 (7.0–20.0%)	14.5 (6.0–20.0%)	0.187
IPQ-Illness representation–Emotional Representations	19.0 (9.0–32.0) ^a,b^	18.0 (9.0–30.0) ^a^	17.0 (12.0–31.0) ^a^	23.0 (8.0–34.0) ^b^	0.001
IPQ-Causal representation–Psychological Attributions	17.0 (8.0–24.0) ^a^	15.0 (8.0–21.0) ^b^	14.0 (10.0–22.0) ^b^	17.0 (8.0–22.0) ^a^	0.001
IPQ-Causal representation–Risk Factors	16.0 (10.0–24.0) ^a^	17.0 (8.0–25.0) ^a^	17.0 (10.0–23.0) ^a^	14.0 (10.0–21.0) ^b^	0.001
IPQ-Causal representation–Immunity	7.5 (4.0–14.0) ^a^	6.0 (3.0–11.0) ^b^	6.0 (3.0–12.0) ^b^	8.0 (3.0–12.0) ^a^	0.001
IPQ-Causal representation–Accident or Chance	7.0 (2.0–10.0%)	7.0 (2.0–10.0%)	6.0 (3.0–10.0%)	7.0 (3.0–10.0%)	0.332

* Kruskal–Wallis test. ^a,b^ Group where the difference originated.

**Table 4:** Correlation of scale scores.

		BDI	BAI
BAI	r	0.848	
	*p*	<0.001	
IPQ-Illness representation–Timeline acute/chronic	r	131	136
	*p*	0.032	0.026
IPQ-Causal representation–Psychological attributions	r	169	−0.071
	*p*	0.005	0.247
IPQ-Causal representation–Risk factors	r	194	0.113
	*p*	0.001	0.063
IPQ-Causal representation–Accident or chance	r	−0.106	125
	*p*	0.081	0.040

**Table 5:** Independent predictors of moderete–severe anxiety level.

	Unadjusted		Adjusted	
Risk Factors	OR (95% CI)	*p*	OR (95% CI)	*p*
Beck Depression Inventory	1.45 (1.30–1.62)	<0.001	1.48 (1.31–1.68)	<0.001
IPQ-Illness representation–Emotional representations	1 (0.96–1.1)	0.41	1.12 (1.10–1.25)	0.035
Hospitalization for cancer	0.47 (0.17–1.28)	0.14	0.20 (0.4–1.1)	0.60
IPQ-Illness representation–Consequences	1.06 (0.99–1.12)	0.06		
IPQ-Illness representation–Timeline cyclical	0.94 (0.86–1.03)	0.17		
IPQ-Causal representation–Accident or chance	0.91 (0.78–1.07)	0.25		

IPQ, Illness Perception Questionnaire; OR, odds ratio; 95% CI, 95% confidence interval. The *p* value of the Hosmere–Lemeshow test was 0.903.

## Data Availability

The datasets used and analyzed during the current study are available from the corresponding author upon reasonable request.
